# Supplementary material for: Downregulation of S1P Lyase Improves Barrier Function in Human Cerebral Microvascular Endothelial Cells Following an Inflammatory Challenge
Source: Int J Mol Sci. 2020 Feb 13;21(4):1240. doi: 10.3390/ijms21041240 (PMC7072972; doi:10.3390/ijms21041240)
Supplement: Supplementary file 1 [file ijms-21-01240-s001.pdf]

Supplementary data

Stepanovska et al.:

Chemicals and reagents: Tris, Tris-HCl, glycin, sodium chloride (NaCl), glycerol, Triton X100, EDTA, sodium dodecyl sulfate (SDS), HEPES, chloroform, isopropanol, Tween®20 and Roti®-Quant were obtained from Carl Roth GmbH (Karlsruhe, Germany). Sodium fluoride (NaF), sodium orthovanadate (Na_3VO_4), sodium pyrophosphate, phenylmethylsulfonyl fluoride (PMSF), DL-dithiothretiol (DTT), β-glycerol phosphate, ethanol, gelatin from porcine skin, L-ascorbic acid, phosphate buffered saline (PBS), lipopolysaccharide (LPS, E. coli O111:B4), KiCqStart™ SYBR® Green qPCR ReadyMix™, cOmplete™ protease inhibitor cocktail, fatty acid- and globulin-free bovine serum albumin (BSA), BSA fraction V, puromycin, dimethyl sulfoxide (DMSO), human fibronectin and horse serum, were from Sigma-Aldrich Chemie GmbH (Buchs, Switzerland). EGTA was from Merck KGaA (Darmstadt, Germany); methanol from Grogg Chemie AG (Stettlen, Switzerland); hydrocortisone from Calbiochem Corp. (La Jolla, CA, USA); RNA-Solv® reagent from Omega Bio-tek Inc., (Norcross, GA, USA); First Strand cDNA Synthesis kit from Thermo Scientific (Waltham, MA, USA). S1P, sphingosine were from Avanti Polar Lipids Inc. (Alabaster, AL, USA), recombinant human tumor necrosis factor α (TNFα), interleukin (IL) -1β and IL-6, interferon (INF) –γ, and recombinant human basic fibroblast growth factor (bFGF) were from PeproTech Inc. (Rocky Hill, CT, USA), the murine collagen IV was from BD Biosciences (Franklin Lakes, NJ, USA) and the Hank's balanced salt solution was from Biochrom (Berlin, Germany). IRDye® 800CW secondary antibodies were bought from LI-COR (Lincoln, NE, USA), and ECL™ HRP-linked antibodies, as well as ECL™ Western Blotting Detection Reagents were obtained from GE Healthcare Limited (Buckinghamshire, UK). Trypsin-EDTA 0.25%, DMEM containing Glutamax™, pyruvate and 4.5 g/L D-glucose and CD lipid concentrate were from Gibco® by Life Technologies Limited (Paisley, UK). Fetal bovine serum (FBS) was purchased from PAN-Biotech GmbH (Catalogue No. P40-37, Aidenbach, Germany). All oligonucleotide primers were from Eurofins Genomics GmbH (Ebersberg, Germany).

Antibodies used: Commercially available antibodies used were: phospho-Ser^{473}Akt (Cell Signaling 4060, 1:500), Akt (Cell Signaling 4691, 1:2000), phospho-Thr^{172} AMPKα (Cell Signaling 2535, 1:1000), AMPKα (Cell Signaling 5831, 1:1000), β-catenin (Transduction Laboratories C19220, 1:1000), GAPDH (Cell Signaling 2118, 1:3000), (acetyl-histone H3 (Cell Signaling 9649, 1:1000), histone H3 (Cell Signaling 4499, 1:1000), ICAM-1 (Cell Signaling 4915, 1:1000), phospho-Thr^{183}/Tyr^{185} SAPK/ JNK (Cell Signaling 9251, 1:1000), phospho-Ser^{63} c-Jun II (Cell Signaling 9261, 1:1000), c-Jun (R&D Systems MAB2670, 1:1000), JunB (Novus NBP1-89544, 4 μg/10 ml), KLF2 (R&D Systems MAB5466, 11 μg/10 ml), KLF4 (R&D Systems AF3640, 5 μg/10 ml), phospho-Thr^{180}/Tyr^{182} p38-MAPK (Cell Signaling 9211, 1:1000), p38-MAPK (Santa Cruz (C-20) sc-535, 1:500), phospho-Thr^{202}/Tyr^{204} p42/p44-MAPK (Cell Signaling 4377, 1:1000), phospho-Ser^{152}/Ser^{156} MARCKS (Cell Signaling 2741, 1:1000), phospho-Ser^{536} p65-NFκB (Cell Signaling 3033, 1:1000), p65-NFκB (Cell Signaling 4764P, 1:1000), p120 (Santa Cruz (6H11) sc-23873 1:500), PECAM-1 (Cell Signaling 3528, 1:1000), phospho-PKC substrate (Cell Signaling 2261, 1:1000), α-tubulin (Sigma, T9026 1:3000), VCAM-1 (Sigma SAB-1406579, 1:1000), VE-cadherin (Santa Cruz sc-9989, 1:500) and ZO-1 (Transduction Laboratories Z72720, 1:1000). IRDye® or HRP-linked secondary antibodies were used and bands were detected and analyzed by the LI-COR Imaging system. Signal intensity was evaluated by ImageJ (National Institutes of Health, MA, USA).

Primer sequences for qPCR analysis: For qPCR, the following primers were used: AXIN2 forward: CGC CAA CGA CAG TGA GAT, reverse: TGC CCA CAC GAT AAG GAG; β-catenin forward: GCG CCA TTT TAA GCC TCT CG, reverse: CCT CAG ACC TTC CTC CGT C; IL-6 forward: TGCAATAACCACCCCTGACC; reverse: CCCAGTGGACAGGTTTCTGA; IL-8 forward: AAGAAACCACCGGAAGGAAC, reverse: ACTCCTTGGCAAAACTGCAC; KLF2 forward: ACCAGTCACAGTTTGGGAGGG, reverse: GCACGCACACAGGTGAGAAG; KLF4 forward: GCCACCCACACTTGTGATT, reverse: TCCACTCACAAGATGACTCAGT; SK-1 forward: GCTTCCTTGAACCATTATG, reverse: TCTCTAGGTCCACATCAG; SK-2 forward:

TCAACCTCATCCAGACAGAACGAC, reverse: CATCCCACTCACTCAGGCTCAG; SPL forward: ATAGATCCTGTCCCTGAAGT, reverse: CACCTTTCACCCGGAAATCA. Primer sequences of human ICAM-1, VCAM-1, PECAM-1, VE-cadherin, MCP-1 and 18S RNA were as previously described [1-3].

Generation of polyclonal antibodies: A polyclonal antibody against human SPL was generated by Eurogentec S.A. (Seraing, Belgium) by synthesizing two peptides (ISADTHKYGYAPKGSC and CTVTQGSQMNGSPKPH), based on the sequence of human SPL (accession number: NM_003901), coupling them to keyhole-limpet hemocyanin and immunizing a rabbit according to standard protocols. The antibodies were purified by affinity chromatography using an anti-peptide-coupled sepharose column. The purified antibody (no.69) was used in a dilution of 1:500. Polyclonal antisera against p42-MAPK and p44-MAPK were generated by immunizing rabbits with two synthetic peptides of each enzyme as previously described and used in a dilution of 1:3000 [4]. Polyclonal rabbit anti-human SK-1 (No. 64, 1:1000) and SK-2 (No. 66, 1:1000) were generated as explained previously by Döll et al. 2005. [5] and Schwalm et al. 2015 [6].

HDAC activity ELISA: In situ Histone Deacetylase (HDAC) Activity Fluorometric Assay Kit from Sigma (Sigma-Aldrich Chemie GmbH, Buchs, Switzerland) has been used to determine overall HDAC activity. Cells were seeded in a 96-well plate, with a density of 15,000 cells/100 µl growth medium and incubated for 24 h. Medium was removed and 100 µl of a reaction mix containing an HDAC substrate with an acetylated lysine side chain was added to the cells for 3 h, following the protocol. Thirty minutes after the addition of the developer that cleaves the deacetylated HDAC substrate, fluorescence was measured on the recommended wavelength and HDAC activity was calculated corrected with the background.

Supplementary references:

1. Imeri, F., et al., *Novel oxazolo-oxazole derivatives of FTY720 reduce endothelial cell permeability, immune cell chemotaxis and symptoms of experimental autoimmune encephalomyelitis in mice.* Neuropharmacology, 2014. **85**: p. 314-27.
2. Imeri, F., et al., *Sphingosine kinase 2 deficient mice exhibit reduced experimental autoimmune encephalomyelitis: Resistance to FTY720 but not ST-968 treatments.* Neuropharmacology, 2016. **105**: p. 341-350.
3. Blanchard, O., et al., *Downregulation of the S1P Transporter Spinster Homology Protein 2 (Spns2) Exerts an Anti-Fibrotic and Anti-Inflammatory Effect in Human Renal Proximal Tubular Epithelial Cells.* International Journal of Molecular Sciences, 2018. **19**(5).
4. Huwiler, A. and J. Pfeilschifter, *Transforming growth factor beta 2 stimulates acute and chronic activation of the mitogen-activated protein kinase cascade in rat renal mesangial cells.* FEBS Lett, 1994. **354**(3): p. 255-8.
5. Döll, F., J. Pfeilschifter, and A. Huwiler, *The epidermal growth factor stimulates sphingosine kinase-1 expression and activity in the human mammary carcinoma cell line MCF7.* Biochimica et Biophysica Acta (BBA)-Molecular and Cell Biology of Lipids, 2005. **1738**(1-3): p. 72-81.
6. Schwalm, S., et al., *Sphingosine kinase 2 deficiency increases proliferation and migration of renal mouse mesangial cells and fibroblasts.* Biological chemistry, 2015. **396**(6-7): p. 813-825.

Supplementary results:

Sphingolipid species (pg/1,5x10⁶ cells)	ctrl		SPL-kd	
	**-**	**TNFα**	**-**	**TNFα**
Sphingosine	4285±382.7	5838±495.1	5387±517.1	6868±244.5
Dihydro-sphingosine	2608±115.1	3435±30.85	1390±166.2	2596±47.40
Sphingosine d20:1	<10			
Sphingosine d20:0	<10			
Ceramide d18:0/16:0	>24000	>24000	7944±3152	19814±1014
Ceramide d18:0/18:0	2548±200.4	4927±317.9	435.4±108.7	1220±83.16
Ceramide d18:0/18:1	<150			
Ceramide d18:0/24:0	6290±448.0	15340±1472	1929±535.7	4138±228.0
Ceramide d18:0/24:1	9222±243.0	16391±606.2	1962±405.9	5068±229.6
Ceramide d18:1/14:0	>6000			
Ceramide d18:1/16:0	>24000			
Ceramide d18:1/18:0	9735±308.4	12906±531.4	2930±645.7	4050±390.5
Ceramide d18:1/18:1	<800	961.8±60.10	<800	<800
Ceramide d18:1/20:0	2954±217.0	4177±207.8	657.9±125.9	960.4±79.52
Ceramide d18:1/22:0	26684±553.5	36560±2416	7072±1038	10033±446.8
Ceramide d18:1/24:0	130926±2599	171609±13529	59269±8916	68499±1819
Ceramide d18:1/24:1	137024±7556	184048±16598	45987±5362	66118±7502
GlcCer d18:1/16:0	191988±19757	158313±31890	119699±9516	104932±6709
GlcCer d18:1/18:0	>6000	>6000	3150±248.8	3291±463.3
GlcCer d18:1/18:1	340.4±129.5	365.9±132.6	200.7±48.96	157.6±19.94
GlcCer d18:1/24:1	71886±10194	76365±5037	49756±2032	43118±4663
LacCer d18:1/16:0	87406±3128	88060±6319	23556±1879	19348±2175
LacCer d18:1/18:0	1890±47.44	1903±416.7	693.3±145.8	561.7±22.01
LacCer d18:1/18:1	<150			
LacCer d18:1/24:0	>60000	>60000	22959±3128	20068±1835
LacCer d18:1/24:1	44919±2430	42946±3923	17067±2251	14847±1779

Table S1: LC-MS/MS results of diverse sphingolipid species. Confluent control (ctrl) and SPL-kd cells were rendered serum-free for 24 h prior to stimulation for 24 h with either vehicle (-) or 1nM TNFα. Lipids were extracted and quantified by LC-MS/MS as described in the Methods section. Results are depicted as picograms per 1,5x10⁶ cells and are means ± S.D. (n=3). GlcCer=glycosylceramide; LacCer=lactosylceramide.

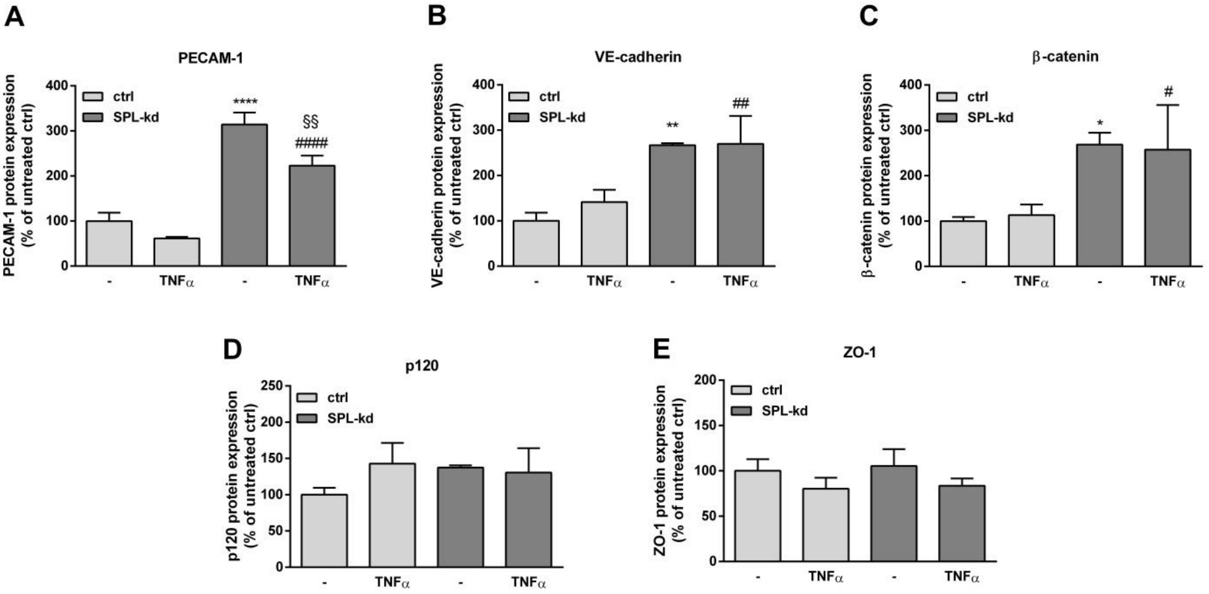

Fig. S1: Effect of SPL knockdown on the expression of adherens junction molecules in TNFα-stimulated HCMECs. Blots shown in Fig. 4 were evaluated by ImageJ software and results for PECAM-1 (B), VE-cadherin (C), β-catenin (D), p120 (E), and ZO-1 (F) were expressed as % of control transduced cells. Data are presented as means ± S.D. (n=3; *p<0.05, **p<0.01, ****p<0.0001 compared to the vehicle-treated controls; #p<0.05, ##p<0.01, ####p<0.0001 compared to the TNFα-treated controls; §§p<0.01 compared to the vehicle-treated SPL-kd).

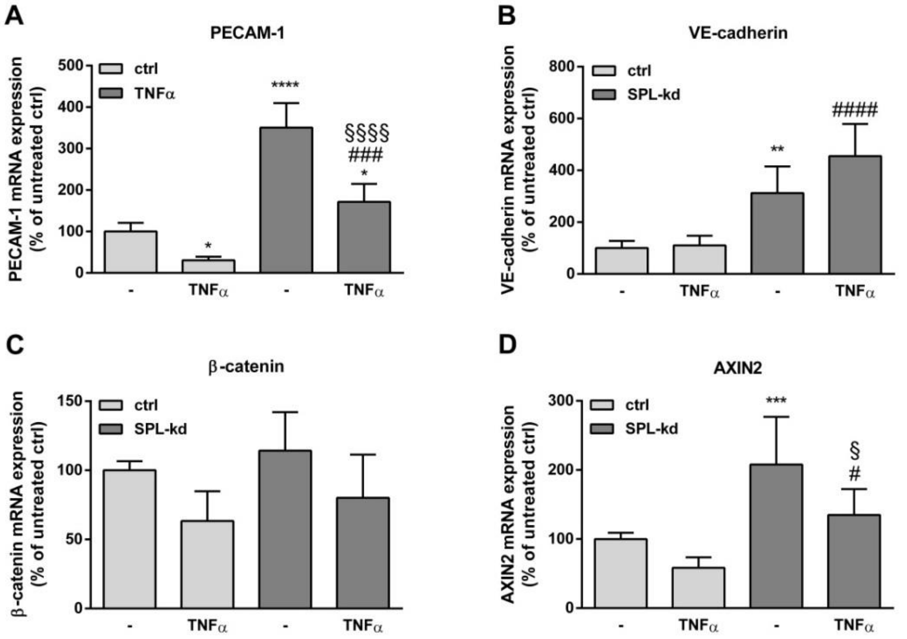

Fig. S2: Effect of SPL knockdown on the mRNA expression of adherens junction molecules in TNFα-stimulated HCMECs. Confluent control (ctrl) and SPL-kd cells were rendered serum-free for 4 h prior to stimulation for 24 h with either vehicle (-) or 1nM TNFα in DMEM/0.1%FBS. Thereafter, RNA was extracted and taken for qPCR analysis of PECAM-1 (A), VE-cadherin (B), β-catenin (C) and AXIN2 (D) mRNA levels. Results are expressed as % of control transduced cells and are means ± S.D. (n=4-6; *p<0.05, **p<0.01, ***p<0.001, ****p<0.0001 compared to the vehicle-treated control; #p<0.05, ###p<0.001, ####p<0.0001 compared to the TNFα-treated control; §p<0.05, §§§§p<0.0001 compared to the vehicle-treated SPL-kd).

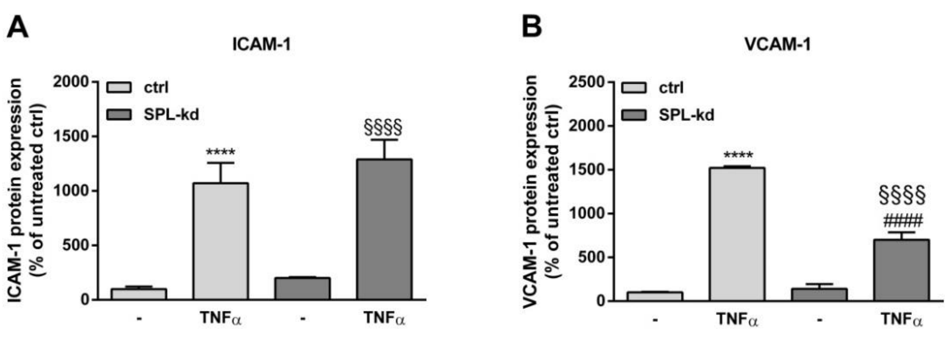

Fig. S3: Effect of TNFα stimulation on the protein expression of adhesion molecules in control and SPL-kd HCMECs. Blots shown in Fig. 5 were evaluated by ImageJ software and results for ICAM-1 (A) and VCAM-1 (B) were expressed as % of control transduced cells. Data are presented as means ± S.D. (n=3****p<0.0001 compared to the vehicle-treated control; ####p<0.0001 compared to the TNFα-treated control; §§§§p<0.0001 compared to the vehicle-treated SPL-kd).

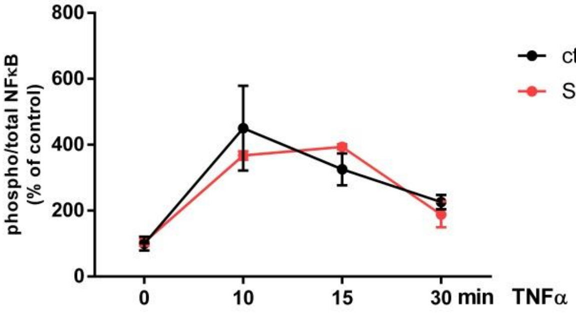

Fig. S4: No alteration of TNFα-stimulated NFκB phosphorylation in SPL-kd cells. Confluent control (ctrl, black circles) or SPK-kd (red circles) HCMEC/D3 cells were rendered serum-free for 4 h prior to stimulation with 1 nM TNFα in serum-free DMEM for the indicated time points. Cells were lysed and harvested for Western blot analysis. Membranes were probed with phospho-(Ser[536]) p65-NFκB and total-p65-NFκB. Bands were evaluated by ImageJ software and the ratio between phospho- and total-NFκB was calculated. Results are expressed as % of control transduced cells and are means ± S.D. (n=3).

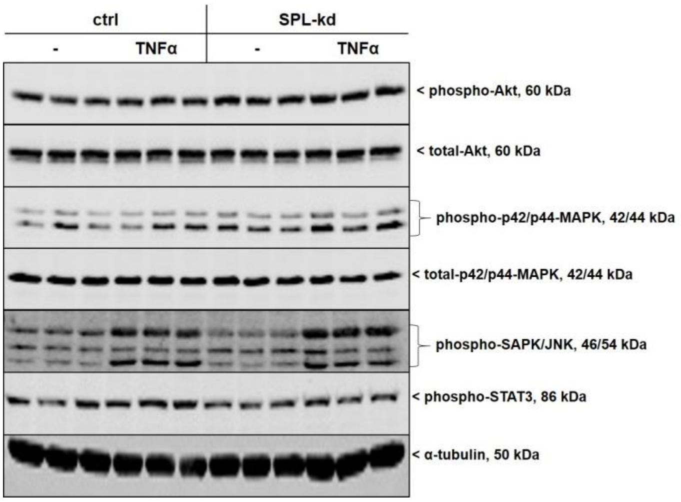

Fig. S5: No effect of SPL-kd on Akt, p42/p44-MAPK, JNK/SAPK and STAT3 signaling pathways in HCMEC/D3 cells. Confluent control (ctrl) and SPL-kd cells were stimulated for 10 minutes with either vehicle (-) or 1nM TNFα. Protein lysates were separated by SDS-PAGE, transferred to nitrocellulose membrane and subjected to Western blot analysis using antibodies against phospho-Akt, total Akt, phospho-p42/p44-MAPK, total p42/p44, phospho-SAPK/JNK, phospho-STAT3 and α-tubulin. Data show representative blots, out of 3-4 independent experiments, performed in triplicates.

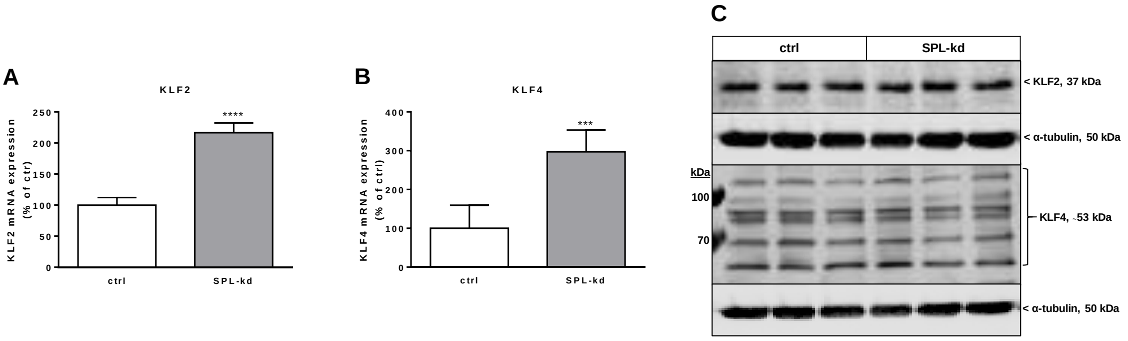

Fig. S6: Effect of SPL-kd on KLF2 and KLF4 mRNA and protein expression. Confluent control (ctrl) and SPL-kd HCMEC/D3 cells were rendered serum-free for 4 h. RNA was extracted and taken for qPCR analysis of KLF2 (A) and KLF4 (B) mRNA expression. Results are expressed as % of control transduced cells and are depicted as means ± S.D. (n=6; ***$p<0.001$, ****$p<0.0001$ compared to the control values). (C) Protein extracts were separated by SDS-PAGE, transferred to nitrocellulose membrane and subjected to Western blot analysis using antibodies against KLF2, KLF4 and α-tubulin. Data show representative blots, out of 2-3 independent experiments, performed in triplicates.

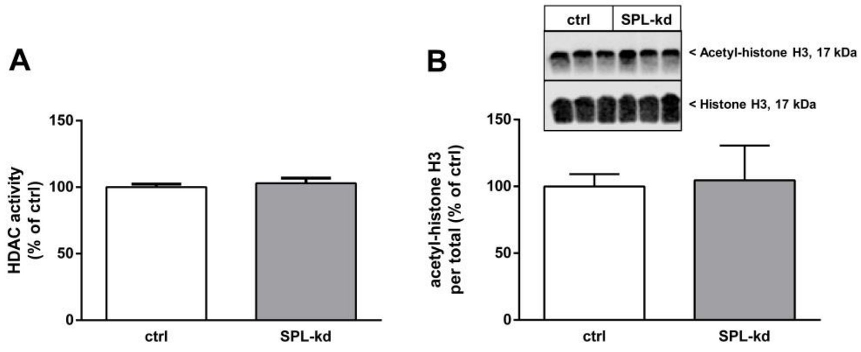

Fig. S7: No effect of SPL-kd on cellular HDAC activity and histone H3 acetylation in HCMECs. (A) Control (ctrl) and SPL-kd HCMEC/D3 cells were seeded in 96-well plates and after 24 h, reaction mixture from the *in situ* HDAC activity fluorometric assay kit (Sigma Aldrich) was added and processed as described in the supplementary Methods section. (B) Confluent control (ctrl) and SPL-kd cells were rendered serum free for 18 h and then taken for protein extraction, SDS-PAGE, and Western blot analysis using antibodies against acetyl-histone H3 (upper panel) and total histone H3 (lower panel). Results in A and B are expressed as % of control transduced cells and are depicted as means ± S.D. (n=3).

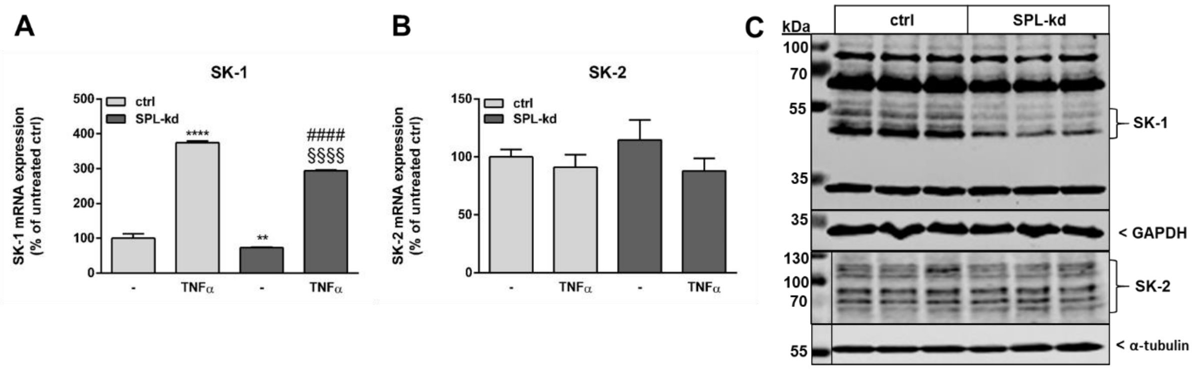

Fig. S8: Effect of SPL-kd on SK-1 and SK-2 mRNA and protein expression. Confluent control (ctrl) and SPL-kd cells were rendered serum-free for 4 h prior to stimulation for 24 h with either vehicle (-) or 1nM TNFα in DMEM/0.1%FBS. Thereafter, RNA was extracted and taken for qPCR analysis of SK-1 (A) and SK-2 (B) mRNA levels, or taken for protein extraction, SDS-PAGE, and Western blot analysis using antibodies against SK-1 and SK-2 (C). Results in A and B are expressed as % of control transduced cells and are means ± S.D. (n=3; **p<0.01, ****p<0.0001 compared to the vehicle-treated control; ####p<0.0001 compared to the TNFα-treated control; §§§§p<0.0001 compared to the vehicle-treated SPL-kd). Results in C show representative blots, out of 2-3 independent experiments, performed in triplicates.

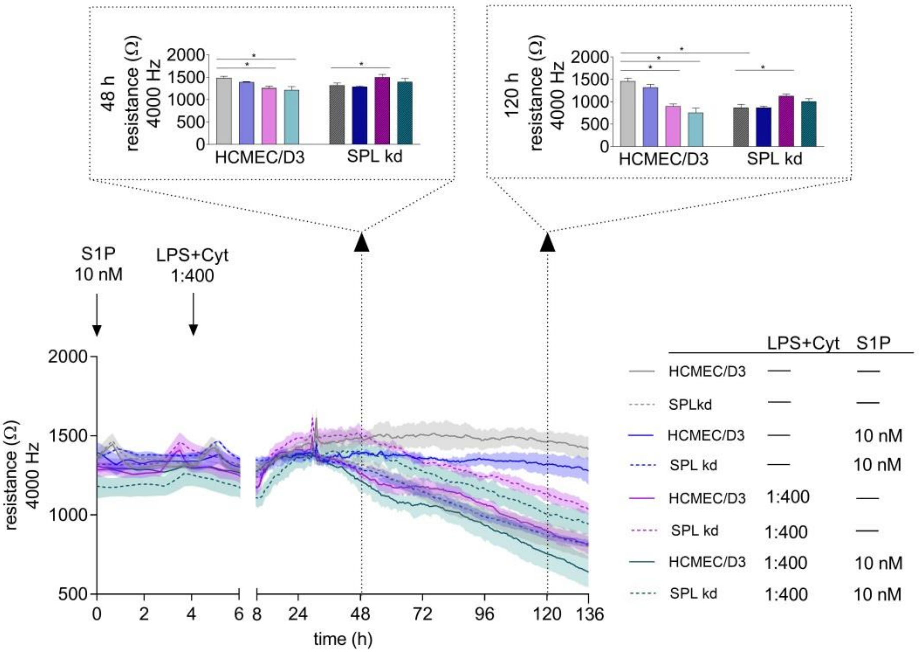

Fig. S9: Effects of pre-treatment with 10 nM S1P on barrier integrity differs in HCMEC/D3 and SPL-kd cells. (A) After development of a stable barrier, HCMEC/D3 control cells (continuous lines) and SPL-kd cells (dashed lines) were pre-treated with 10 nM S1P (t = 0 h) and stimulated with LPS+Cyt 1:400 (t = 4 h). The two observation time points, 48 h and 120 h (indicated by dotted arrows) were analyzed in detail**.** Data are expressed as mean ± SD (n=3), *p<0.05.

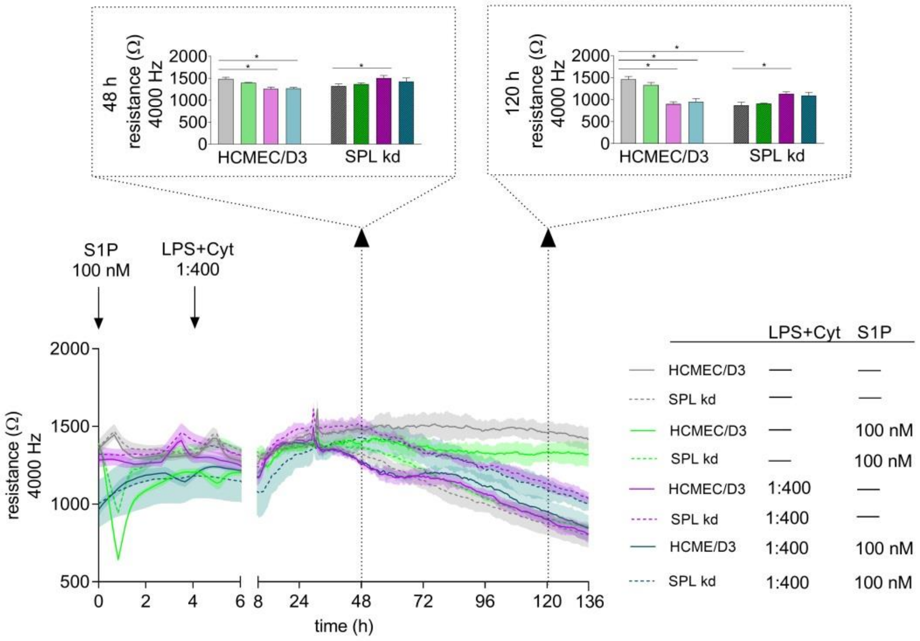

Fig. S10: Effects of pre-treatment with 100 nM S1P on barrier integrity differs in HCMEC/D3 and SPL-kd cells. (A) After the development of a stable barrier, HCMEC/D3 control cells (continuous lines) and SPL-kd cells (dashed lines) were pre-treated with 100 nM S1P (t = 0 h) and stimulated with LPS+Cyt 1:400 (t = 4 h). Resistance values were analyzed at the two observation time points 48 h and 120 h (indicated by dotted arrows). Data are expressed as mean ± SD, n = 3, *p<0.05.

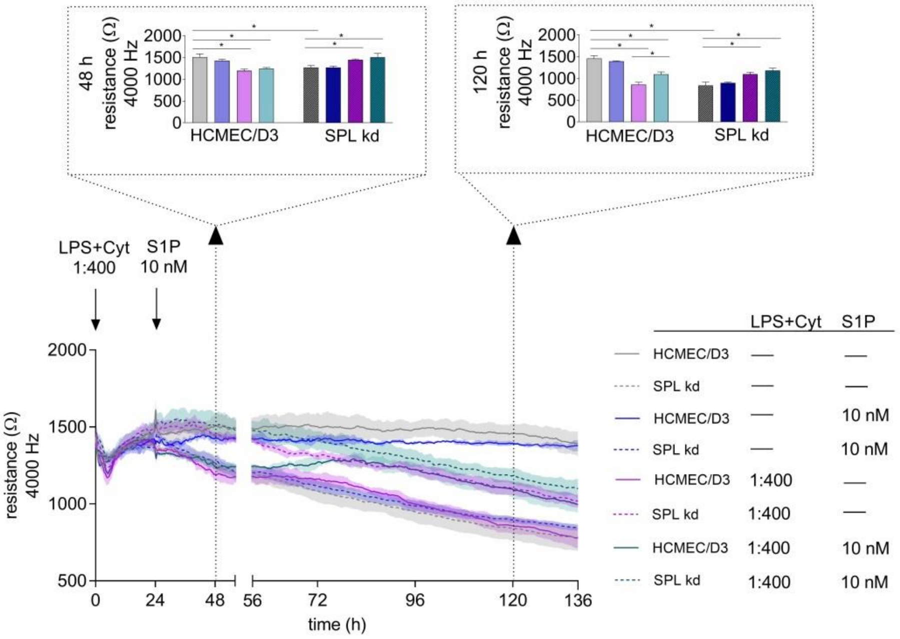

Fig. S11: Effects of post-treatment with 10 nM S1P on barrier integrity differs in HCMEC/D3 and SPL-kd cells. (A) After the development of a stable barrier, HCMC/D3 control cells (continuous lines) and SPL-kd cells (dashed lines) were stimulated with LPS+Cyt 1:400 (t = 0 h) and post-treated with 10 nM S1P (t = 24 h). Resistance was analyzed at the two observation time points 48 h and 120 h (indicated by dotted arrows). Data are expressed as mean ± SD, n = 3, *p<0.05.

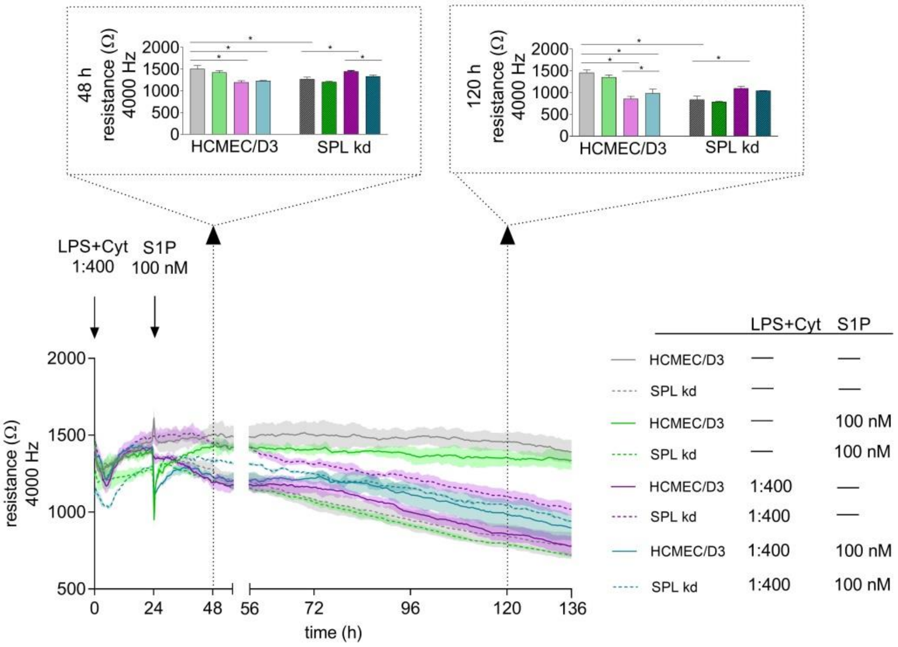

Fig. S12: Effects of post-treatment with 100 nM S1P on barrier integrity differs in HCMEC/D3 and SPL-kd cells. (A) After the development of a stable barrier, HCMEC/D3 control cells (continuous lines) and SPL-kd cells (dashed lines) were stimulated with LPS+Cyt 1:400 (t = 0 h) and post-treated with 100 nM S1P (t = 24 h). Resistance was analyzed at the two observation time points 48 h and 120 h (indicated by dotted arrows). Data are expressed as mean ± SD, n = 3, *p<0.05.
